# Nerve Growth Factor Shows Biphasic Expression during Adjuvant-Induced Neurogenic Inflammation

**DOI:** 10.3390/ijms25074029

**Published:** 2024-04-04

**Authors:** Vikramsingh Gujar, Radhika D. Pande, Subhas Das

**Affiliations:** 1 Department of Anatomy and Cell Biology, Oklahoma State University, Center for Health Sciences, Tulsa, OK 74107, USA; 2 Department of Biochemistry and Microbiology, Oklahoma State University, Center for Health Sciences, Tulsa, OK 74107, USA; radhika.pande@okstate.edu (R.D.P.); subhas.das@okstate.edu (S.D.)

**Keywords:** inflammation, nerve growth factor (NGF), neurogenic inflammation, acute inflammation, nociception, NGF-TrkA signaling, biphasic

## Abstract

Chronic inflammatory diseases are considered the most significant cause of death worldwide. Current treatments for inflammatory diseases are limited due to the lack of understanding of the biological factors involved in early-stage disease progression. Nerve growth factor (NGF) is a neurotrophic factor directly associated with inflammatory and autoimmune diseases like osteoarthritis, multiple sclerosis, and rheumatoid arthritis. It has been shown that NGF levels are significantly upregulated at the site of inflammation and play a crucial role in developing a robust inflammatory response. However, little is known about NGF’s temporal expression profile during the initial progressive phase of inflammation. This study aimed to determine the temporal expression patterns of NGF in rat skin (epidermis) during adjuvant-induced arthritis (AIA). Sprague Dawley rats were randomly divided into control and complete Freund’s adjuvant (CFA)-treated groups. Levels of NGF were evaluated following unilateral AIA at different time points, and it was found that peripheral inflammation due to AIA significantly upregulated the expression of NGF mRNA and protein in a biphasic pattern. These results suggest that NGF signaling is crucial for initiating and maintaining peripheral neurogenic inflammation in rats during AIA.

## 1. Introduction

Nerve growth factor (NGF) is a neurotrophin for a subset of nociceptive sensory neurons and a protein that modulates the differentiation of developing peripheral neurons [1]. During inflammation generated by the unilateral injection of complete Freund’s adjuvant (CFA), the level of NGF is elevated in the skin [2]. NGF is also increased in various animal models mimicking inflammation, including carrageenan and formalin [3,4]. However, the expression pattern of NGF during the progressive phase of peripheral inflammation has not yet been well defined.

Pain and inflammation are considered debilitating, but at the same time, they are also the protective responses necessary for survival. Pain is the mechanism that provides information about the presence or threat of an injury [5]. In addition to the afferent function, such as conveying information to the central nervous system, the dorsal root ganglion (DRG) primary sensory neurons, specifically C and some Aδ fibers, regulate the vascular and tissue function at their peripheral targets. For example, during neurogenic inflammation, tissue damage causes the depolarization of the primary afferents, which releases neuropeptides such as substance P (SP), calcitonin gene-related peptides (CGRPs), and neurotransmitters such as glutamate, leading to increased vascular permeability and plasma extravasation [6]. Due to this increased vascular permeability, the inflammation site recruits inflammatory cells such as T lymphocytes, mast cells, macrophages, and neutrophils [7]. These cells change the chemical milieu at the site of injury/inflammation by releasing pro-inflammatory molecules such as prostaglandins, bradykinin, histamine, and serotonin. Cytokines such as interleukins (ILs), tumor necrosis factor (TNF), and neurotrophins like NGF are released during inflammation [8].

Inflammation in the periphery stimulates the sensitization of peripheral nerves and changes neuronal cells in the DRG by generating signals that travel in a backward direction in pain-sensing neurons. The retrograde signals activated by NGF in the opposite direction activate or boost the transcription of molecules that promote pain, such as neurotransmitters and neuropeptides, leading to an increase in both central and peripheral sensitization [1,9]. To meet the considerable challenge of conveying information from the periphery to the cell body located far away, dedicated mechanisms of retrograde NGF signaling have evolved, which carry the signals generated from the axonal endings to the neuron’s cell body [10]. Following NGF engagement, TrkA forms a heterodimer and becomes autophosphorylated. This complex is internalized by clathrin-dependent endocytosis, which gives rise to signaling endosomes. It has been shown that NGF signaling endosomes are multivesicular bodies (MVBs) that mediate long-range retrograde transport [11].

Although the levels of NGF are upregulated after a noxious stimulus, the information regarding the expression pattern of NGF during the initial progression of peripheral inflammation is unknown. In this study, we determined the effect of antigen-induced arthritis (AIA) in rat skin (epidermis) on the time-course alteration of the expression of NGF. For this purpose, we employed the unilateral AIA rat model and measured the rats’ hind paw metatarsal thickness to determine the severity of inflammation. We also evaluated the NGF protein and mRNA levels at different time points during the phasic progression of AIA-induced inflammation. 

## 2. Results

### 2.1. Changes in Hind Paw Edema and Body Weight

**Figure 1:**
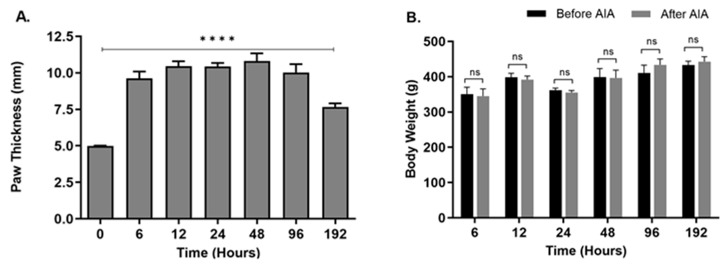
Effect of peripheral inflammation on hind paw edema and body weight. The animals were injected with CFA in the right hind paw, and their paw thickness and body weight were measured using a dial caliper and weighing balance, respectively. (**A**) Hind paw edema by measuring the metatarsal thickness before and after CFA treatment at different time points (**** *p* < 0.0001, compared with controls; *n* = 6). (**B**) The body weight of animals (*n* = 6; Each group) treated with CFA at different time points compared to the control animals (ns; insignificant). Data are shown as the mean ± SEM, *t*-test.

The metatarsal thickness of the ipsilateral posterior paws was significantly increased (*p* < 0.0001) in animals treated with CFA compared to the control animals. The increased thickness was observed at all seven time points, with the highest peak at 48 h (Figure 1A), suggesting a robust inflammatory response. An increase in the metatarsal thickness of rats suggests the presence of inflammation. This observation is consistent with known physiological reactions, where inflammation can cause tissue swelling by increasing blood vessel permeability and the buildup of immune cells and inflammatory substances at the inflamed location [12]. In the experiment assessing the impact of increasing peripheral inflammation on NGF expression in rat epidermis, the rise in metatarsal thickness acts as a supporting sign of the inflammatory reaction caused by AIA. The increased thickness in the metatarsal region indicates a specific swelling of tissues, perhaps caused by the inflammatory process initiated by AIA. Measuring the metatarsal thickness assists in determining the efficiency of the experimental model in replicating inflammatory conditions and highlights the need to investigate NGF expression in relation to inflammation-related diseases such as pain sensitivity and tissue damage. The body weight of animals after CFA treatment was not significantly different from the control animals at any of the seven time points (Figure 1B), suggesting that the animals did not experience significant distress as a direct result of the injection. This observation is crucial in assessing the welfare and well-being of the experimental animals throughout this study. Significant changes in body weight, such as a decrease, could indicate distress, discomfort, or adverse effects associated with the injection procedure or subsequent inflammatory response. However, the stable body weight suggests that the rats maintained their physiological equilibrium and were not adversely affected by the CFA injection in terms of their overall health and nutritional status during the monitored period. This finding provides important reassurance regarding the ethical conduct of the study and supports the interpretation of experimental results obtained from the rats subjected to CFA-induced inflammation.

### 2.2. Change in NGF-Immunoreactivity (-ir) during Inflammation

**Figure 2:**
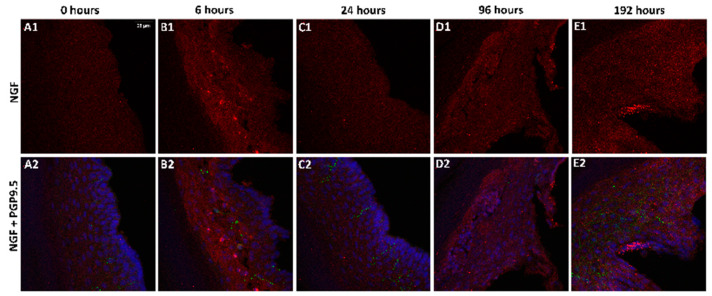
Effect of peripheral inflammation on the immunoreactivity of NGF. Double-labeling immunofluorescence with NGF and PGP 9.5 shows the expression of NGF in epidermal tissue at different time points. Representative images of rat epidermis at 0 h (**A1**,**A2**), 6 h (**B1**,**B2**), 24 h (**C1**,**C2**), 96 h (**D1**,**D2**), and 192 h (**E1**,**E2**) after CFA treatment. (**A1**–**E1**) NGF fluorescence (red); (**A2**–**E2**). Merged images of NGF (red), PGP 9.5 (green), and DAPI (blue). Scale bar = 25 μm is applied to all images, *n* = 6.

**Figure 3:**
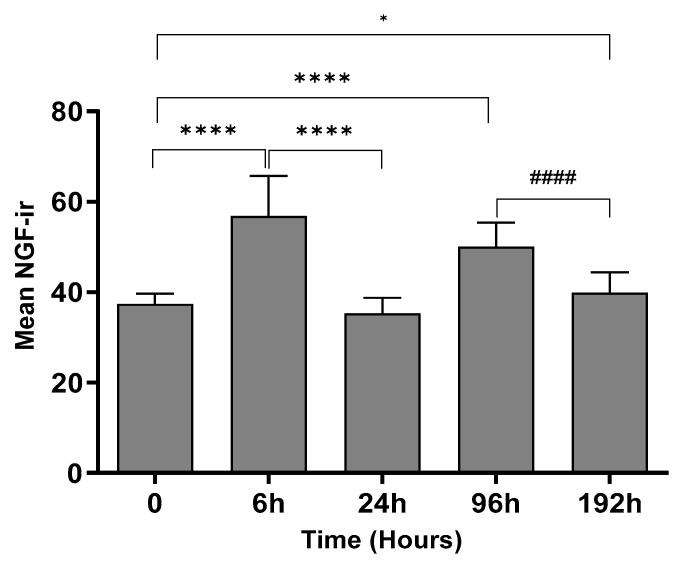
Effect of peripheral inflammation on the immunoreactivity of NGF. Quantitative image analysis shows that NGF-ir in the epidermal tissue was significantly higher after 6 h (**** *p* < 0.0001), 96 h (**** *p* < 0.0001), and 192 h (* *p* = 0.0362) of CFA treatment compared to the control animals (0 h). However, this NGF-ir after 192 h was significantly reduced compared to 96 h of inflammation (#### *p* < 0.0001). Data are shown as the mean ± SEM, *n* = 6, *t*-test.

To determine the levels of the NGF protein, the double labeling of NGF and PGP9.5 was performed on epidermal sections of the rat’s hind paws, and DAPI was used for nuclear staining. The images (Figure 2) indicate that the immunoreactivity of NGF after 6 h and 96 h of inflammation was comparatively higher than the control animals. The results from quantitative image analysis (Figure 3) indicate that the NGF immunoreactivity was considerably higher after 6 h of inflammation (*p* < 0.0001) with respect to the control sample (0 h). The immunoreactivity was reduced after 24 h (*p* < 0.0001) but increased significantly after 96 h of treatment (*p* < 0.0001) and further decreased after 192 h (*p* = 0.036) compared to 0 h, indicating a biphasic response. After 192 h, the immunoreactivity was found to be lower compared to the 96 h sample (*p* < 0.0001). PGP 9.5 immunoreactivity in intraepidermal nerve fibers was qualitatively similar at all time points and similar to the results in our other studies [13,14].

### 2.3. NGF Expression Shows Biphasic Response during Peripheral Inflammation

**Figure 4:**
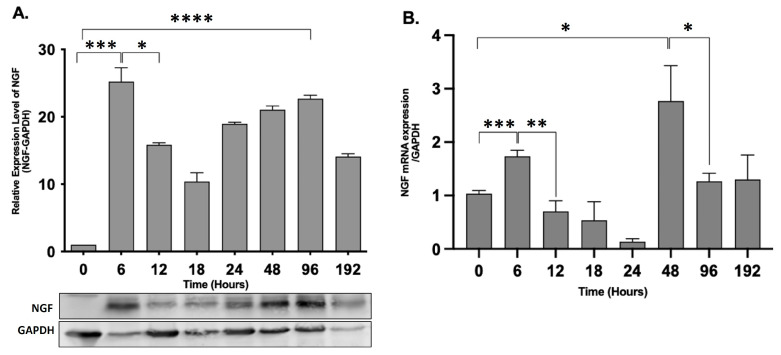
NGF expression shows biphasic response during peripheral inflammation in rat epidermis. (**A**) The Western blot analysis of NGF protein expression under control and AIA conditions (**** *p* < 0.0001). The data were normalized against the GAPDH housekeeping protein and represented as relative change. (**B**) Comparison of NGF mRNA expression in control and CFA-treated animals by qPCR at different time points. The levels of NGF mRNA were significantly upregulated after 6 h of AIA (*** *p* = 0.0007), with a decrease after 12 h (** *p* = 0.0015) and a second spike after 48 h (* *p* = 0.0109) Data were analyzed with the ∆∆Ct method and represented as the fold change against GAPDH. Bars represent means ± SD, *t*-test (*n* = 3).

To evaluate the effect of progressive peripheral inflammation on NGF expression in rat epidermis, we determined the expression of the NGF protein and mRNA by performing Western blot analysis and qPCR, respectively. The levels of NGF mRNA were significantly upregulated after 6 h of AIA (*p* = 0.0007), with a decrease after 24 h and a second spike after 48 h (*p* = 0.0109) (Figure 4B). The NGF protein level showed a similar biphasic response with two peaks after 6 h (*p* = 0.0003) and 96 h (*p* < 0.0001) of peripheral inflammation compared with the control animals (0 h) (Figure 4A).

## 3. Discussion

The unilateral AIA caused a significant increase in the metatarsal thickness of the ipsilateral hind paws, indicating a robust inflammatory process, as reported previously [15,16]. The injection of CFA induces the release of several inflammatory mediators, leading to peripheral and central sensitization [17], confirming that the AIA rat model is appropriate for studying the process of the acute and chronic inflammatory process [18]. The present study corroborates the contribution of NGF in developing and maintaining peripheral inflammation. We found that the levels of the NGF protein were significantly upregulated after six hours of noxious stimulus and decreased after twenty-four hours. The levels again increased after ninety-six hours, showing a biphasic response. 

As inflammation is converted from acute to chronic, it maintains its distinct characteristics, such as increased vascular permeability, vasodilation, and macrophage migration [19]. After the initiation of inflammation in the periphery, both central and peripheral nervous systems exhibited significant changes that led to altered sensory inputs and processing, for instance, the enhanced excitability of primary afferent neurons [20]. Skin, especially the epidermis, is densely innervated by specialized nerve endings of sensory afferent neurons that express and release neuropeptides. SP and CGRP-specific receptors are located on the epidermal keratinocytes, and during inflammation, SP can stimulate epidermal keratinocytes to produce and release neurotrophins and inflammatory mediators like NGF and interleukin-1β, respectively [21,22,23,24]. 

In this study, we also confirmed that the mRNA levels of NGF are mirrored with that of the protein levels, showing two peaks. Previous work has shown the instant surge in NGF protein and mRNA levels in different inflammatory models of formalin, complete Freund’s adjuvant, carrageenan, and turpentine oil [2,4,25,26]. The primary source of NGF is attributed to specific inflammatory cells like mast cells [27], macrophages [28], lymphocytes, and eosinophils [29], whereas the source in this study is indicative of epidermal keratinocytes. This modulation of NGF expression during peripheral damage is likely mediated by either neuropeptides released from cutaneous nerves or cytokines typically involved in tissue damage like IL-1β, IL-6, and TNF-α [30,31,32,33,34]. Intraepidermal nerve fibers labeled with PGP 9.5 were consistent across the temporal course of AIA, indicating their potential for promoting and responding to inflammation and their presumptive uptake of the elevated NGF.

The immunohistochemical image analysis and Western blotting showed the two peaks of the NGF protein, but there is a disparity in the percent change levels (immunohistochemical image analysis; 6 h—15%, 96 h—10% and Western blotting; 6 h—30%, 96 h—25% approx). These variations may exist due to the change in the protein conformation and target accessibility in immunohistochemistry compared to Western blotting Although the NGF antibody (E-12) produced by Santa Cruz Biotechnology Inc, Dallas, TX, USA. has specificity for both the mature (13 kDa) and pro-NGF (27 and 35 kDa) forms [35], our Western blotting protocol was only able to detect the pro-forms, and we failed to demonstrate the lower molecular weight mature NGF forms. This might be due to the specific tissue processing, e.g., thermolysin separation, and Western blotting conditions we employed for this study. The pro-NGF is formed by the alternate splicing of the NGF gene, which is cleaved into a mature form [36]. The Western blotting results are in agreement with prior studies on the rat retina during optic nerve crush [37]. We also observed variations in the percentage change in mRNA levels compared to the protein levels. These changes may result from the change in expression levels of the housekeeping gene, which could be due to variations in biological conditions or differences in specific experimental conditions [38,39]. Considering this, we tested several housekeeping genes like β-actin, GAPDH, and 18 s rRNA gene and found that the mRNA expression of GAPDH had the minimum variation in the skin of control and AIA rats. The observed discrepancy between NGF mRNA and protein levels at 24 h suggests intricate dynamics in post-transcriptional regulation and protein synthesis. Despite lower NGF mRNA levels, post-transcriptional processes like mRNA stabilization or microRNA-mediated regulation may enhance translation efficiency or prolong mRNA half-life, leading to higher protein levels. Additionally, a temporal delay between mRNA changes and protein synthesis, along with potential compensatory mechanisms, could maintain NGF protein levels despite reduced mRNA expression. These findings underscore the complexity of NGF regulation and warrant further investigation into underlying molecular mechanisms.

This study presents intriguing insights into the temporal expression profile of NGF in rat skin during adjuvant-induced arthritis (AIA). While using Sprague Dawley rats offers a common animal model for biomedical research, caution must be exercised in extrapolating findings to human inflammatory diseases, given the potential species-specific differences. Additionally, this study’s evaluation of NGF expression at different time points provides valuable temporal data; however, the chosen time points may not fully capture the entire regulatory profile of the NGF, potentially overlooking critical phases of its expression. Furthermore, the study’s focus on NGF expression highlights an essential aspect of neurogenic inflammation. Yet, it does not explore other potential contributors to this process, such as neurotrophic factors or inflammatory mediators. A deeper mechanistic understanding and investigation into the broader molecular landscape could offer a more comprehensive view of the inflammatory cascade. Therefore, while this study sheds light on NGF’s role in AIA, addressing these limitations, including the specificity of the animal model, the comprehensiveness of time point selection, the exploration of additional molecular factors, and the mechanistic insights, can further enhance its relevance and applicability in advancing our understanding of inflammatory diseases. 

## 4. Materials and Methods

### 4.1. Animals 

The research utilized Sprague Dawley rats that were bred on-site and weighed between 250 and 350 g. The rats were exposed to a 12 h cycle of light and dark and had free access to food and water. The study was carried out at the Oklahoma State University-Centre for Health Sciences (OSU-CHS) with the full consent of the OSU-CHS Institutional Animal Care and Use Committee (Protocol Number 2020/01). All steps were taken to limit the number of animals used in the experiments.

### 4.2. Induction of Adjuvant-Induced Arthritis (AIA)

Unilateral hind paw inflammation was induced in rats using complete Freund’s adjuvant (CFA; Millipore Sigma, Burlington, MA, USA). The rats were given isoflurane anesthesia (initially 5%, then lowered to 2.5%) and were then injected with 150 µL of a 1:1 CFA emulsion in 1X phosphate-buffered saline intradermally into the underside of the rats’ right glabrous hind paw. Control animals underwent the same anesthesia procedure and received 150 µL of PBS injection. The inflammation persisted for 6, 12, 24, 48, 96, and 192 h, and the skin was harvested for analysis after euthanization by CO_2_ asphyxiation at each time point. One limitation of using animals with phosphate-buffered saline (PBS) injections, as demonstrated by our lab in previous studies, is the induction of minor inflammation at the injection site [16]. This inflammation could potentially confound the interpretation of the experimental results, particularly when studying phenomena such as neurotrophic factor expression during inflammation. However, to mitigate this limitation, we implemented appropriate normalizing techniques to account for variations in baseline inflammation induced by PBS injection. 

### 4.3. Hind Paw Edema and Body Weight

Metatarsal thickness was used to evaluate the severity of the inflammation in animals. The thickness of the rats’ hind paws on the same side as the inflammation was measured using a dial caliper (Mitutoyo, Kawasaki City, Japan) to the nearest 0.05 mm at each time point prior to tissue collection. The body weight of each animal was also measured using an electronic balance to monitor any significant changes due to ongoing inflammation [40]. 

### 4.4. Thermolysin Treatment

Skin samples were processed for the epidermal–dermal separation, as described previously [14]. Briefly, the skin sample was collected after each time point in Dulbecco’s Modified Eagle’s medium (DMEM; Millipore Sigma, Burlington, MA, USA) on ice. The skin tissues were transferred in a 0.5 mg/mL thermolysin solution (Millipore Sigma) and kept at 4 °C for 2 h. After the incubation, the stratum corneum and the epidermis were separated from the dermis and then immersed in 5 mM of EDTA in DMEM for 30 min to stop the thermolysin activity. Following the proteolytic treatment, the epidermal sections were used for RNA and protein analysis. 

### 4.5. Immunohistochemistry (IHC)

The IHC was performed as described previously [14,41,42]. Briefly, after treating the skin samples with thermolysin, the epidermal layer of the skin was submerged in a solution of 0.96% (*w*/*v*) picric acid and 0.2% (*w*/*v*) formaldehyde in a 0.1 M sodium phosphate buffer at pH 7.3. This immersion lasted for three hours at 4 °C. The tissues were then transferred to a solution of 10% sucrose in PBS at pH 7.3 and kept overnight at 4 °C. The vertical embedding of the epidermal section of the skin was performed in a frozen block, and the resulting block was sliced into 14 μm sections using a Leica CM 1850 cryostat. Each slide was then coated with gelatin with three sections mounted onto it. Five slides were dried for one hour at 37 °C at each time point. The dried sections were rinsed with PBS three times for 10 min each. Primary antibodies, anti-nerve growth factor (Santa Cruz Biotechnology, Dallas, TX, USA) at a 1:1000 dilution, and anti-PGP9.5 (Cedarlane Labs, Burlington, ON, Canada) at a 1:10,000 dilution, were then incubated with the frozen sections for four days at 4 °C. After primary antibody incubation, the sections were rinsed three times with PBS and then incubated with anti-mouse Alexa Flour 555 and anti-rabbit FITC 488 for 60 min at room temperature in a dark box. The sections were then rinsed with PBS once and incubated with 300 nM of 4′,6-diamidino-2-phenylindole (DAPI) diluted in PBS for 15 min at room temperature. After removing the DAPI, the sections were rinsed with PBS three times and mounted using ProLong Gold Mounting Media (Invitrogen, Waltham, MA, USA).

### 4.6. Quantitative Image Analysis

The Leica (Wetzlar, Germany) DMI 4000B confocal microscope with a 40X objective was used to capture the images. The sequential merging of 3–10 confocal images was performed to obtain the final images that represented the field of view. The resultant micrographs were saved in an 8-bit grayscale tiff format with a pixel intensity range of 0–255. Three filters, FITC, TRITC, and DAPI, were used to detect each fluorophore for each field of view from each epidermal section. An area of 7392 μm^2^ was selected as a fixed box to choose the epidermal region of interest (ROI) for each image. ImageJ software (Version - 1.53t) was used to analyze all the images. The ROI manager was used to select and add all the ROIs for a given image. Area and mean gray values were then measured and exported for further statistical analysis [16]. 

### 4.7. RNA Isolation and Quantitative Real-Time PCR

The epidermal tissue of naïve and treated rats at various time points after CFA injection was used to isolate and purify total RNA using Trizol (Thermo Fisher Scientific, Waltham, MA, USA). The M-MLV reverse transcriptase (Promega, Madison, WI, USA) was employed to perform complementary DNA (cDNA) synthesis. Quantitative real-time PCR (qRT-PCR) was carried out using the ABI StepOneTM system from Applied Biosystems (Waltham, MA, USA). To detect NGF mRNA, SYBR Select Master Mix from Thermo Fisher Scientific (Waltham, MA, USA) was utilized, and GAPDH RNA was used as an internal reference for NGF. The primer sequences used for NGF and GAPDH are presented in Table 1.

The PCR analysis results were reported as the threshold cycle (Ct), which determined the mRNA of the target gene in relation to the reference gene. The difference between the number of cycles required to detect the PCR products for the target and reference genes was represented by ΔCt. ΔΔCt was the difference between the naïve animal group and the AIA group. Finally, the relative amount of target mRNA in the CFA-treated sample compared to the control animal group was expressed as 2^−ΔΔCt^.

### 4.8. Western Blot Analysis

After the thermolysin treatment, the epidermal tissue was homogenized with a lysis buffer containing a protease inhibitor cocktail (Millipore Sigma). Samples were centrifuged at 14,000 rpm for 15 min at 4 °C. The supernatant was collected, and the total protein concentration was evaluated using the Pierce™ BCA Protein Assay Kit (Thermo Fisher Scientific, Waltham, MA, USA). Samples (50 µg/mL of total Protein) suspended in 10 mM of Tris Base, 1 mM of EDTA, 2.5% SDS, 5% β-mercaptoethanol, and 0.01% bromophenol blue were heat-denatured at 100 °C for 10 min. Samples were separated using 12% TGX™ FastCast™ gels (Bio-Rad Laboratories, Hercules, CA, USA). Proteins were transferred onto a nitrocellulose membrane (Bio-Rad Laboratories) using the Mini Trans-Blot Cell (Bio-Rad Laboratories). The membranes were then blocked with 5% non-fat dry milk in Tris-buffered saline at room temperature, followed by rinsing with TBST. Overnight incubation at 4 °C with the NGF antibody (E-12, Santa Cruz, Dallas, TX, USA) at a 1:1000 dilution in TBST/5% milk was performed. The membranes were washed with TBST and then incubated in secondary alkaline phosphatase-labeled anti-mouse and anti-rabbit IgG (Promega, Madison, WI, USA) at a 1:1000 dilution for 120 min. The details of the antibody used for Western blot is provided in Table 2. Western blot images were obtained using the ECF substrate on a Typhoon 9410 Variable Mode Imager. Image analysis was conducted using ImageJ (Version 1.53t - National Institute of Health).

### 4.9. Statistical Analysis

Student’s *t*-test was performed on all data sets using GraphPad Prism (version 9, GraphPad Prism). *p* values less than 0.05 were considered significant for all tests. The data presented in the graph are grouped by mean ± SEM (and/or SD). 

## 5. Conclusions

In summary, the present result indicates that the biphasic increase in the expression of the NGF occurs after 6 h and 96 h, hence playing a vital role in the phasic progression of inflammation. This temporal change in NGF expression during peripheral inflammation may help determine the timing of therapeutic interventions like anti-NGF antibodies for treating diseases like osteoarthritis and rheumatoid arthritis. Chronic inflammatory conditions in humans can undergo amplified active and preclinical or quiescent stages [43,44,45,46], and effective anti-NGF therapy may require specific temporal dosing. Further studies are required to assess the NGF levels during chronic inflammation to fully understand the role of NGF signaling during peripheral sensitization and determine novel therapeutic targets. 

## Figures and Tables

**Table 1:** Primer sequences used for quantitative PCR.

Gene	Primer Sequence
Nerve growth factor (NGF)	NGF-F: 5′-GTGGACCCCAAACTGTTTAAGAAACGG-3′ NGF-R: 5′-GTGAGTCCTGTTGAAGGAGATTGTACCATG-3’
GAPDH	GAPDH-F: 5′- GAACCACGAGAAATATGACAACTCCCTCAAG-3′ GAPDH-R: 5′- GCAGTGATGGCATGGACTGTGG-3′

**Table 2:** Details of primary and secondary antibodies used in immunofluorescence and Western blot studies.

	Primary Antibodies	Dilutions	Secondary Antibodies	Dilutions
Immunohistochemistry	NGF Anti-mouse (E-12, Santa Cruz, Dallas, TX, USA)	1:1000	Donkey anti-mouse Alexa Flour 555 (Invitrogen; Carlsbad, CA, USA)	1:1000
	PGP 9.5 Anti-rabbit (Cederlane Labs, Burlington, ON, Canada)	1:10,000	Donkey anti-rabbit FITC 488 (Invitrogen; Carlsbad, CA, USA)	1:1000
Western Blot	NGF Anti-mouse (E-12, Santa Cruz, Dallas, TX, USA)	1:1000	Goat anti-mouse IgG (Promega; Madison, WI, USA)	1:1000
	GAPDH (Santa Cruz, Dallas TX, USA)	1:1000	Goat anti-rabbit IgG (Promega; Madison, WI, USA)	1:1000

## Data Availability

All relevant data are included in the manuscript.
